# *N*-[(2-Chloro-8-methyl­quinolin-3-yl)meth­yl]-4-meth­oxy­aniline

**DOI:** 10.1107/S1600536810041061

**Published:** 2010-10-20

**Authors:** Raouf Boulcina, Nassima Benhamoud, Sofiane Bouacida, Thierry Roisnel, Abdelmadjid Debache

**Affiliations:** a Laboratoire des Produits Naturels d’Origine Végétale et de Synthèse Organique, PHYSYNOR, Université Mentouri–Constantine, 25000 Constantine, Algeria; b Unité de Recherche de Chimie de l’Environnement et Moléculaire Structurale, CHEMS, Faculté des Sciences Exactes, Département de Chimie, Université Mentouri–Constantine, 25000 Algeria; c Département de Chimie, Facult des Sciences Exactes et Sciences de la Nature, Universit Larbi Ben M’hidi, Oum El Bouaghi, Algeria; d Centre de difractométrie X, UMR 6226 CNRS Unité Sciences Chimiques de Rennes, Université de Rennes I, 263 Avenue du Général Leclerc, 35042 Rennes, France

## Abstract

In the title compound, C_18_H_17_ClN_2_O, the quinoline ring system is essentially planar; the r.m.s. deviation for the non-H atoms is 0.04 Å with a maximum deviation from the mean plane of 0.026 (4) Å for the C atom bonded to the –CH_2_– group. The meth­oxy-substituted benzene ring forms a dihedral angle of 70.22 (4)° with this ring system. The crystal structure can be described as zigzag layers in which the quinoline ring systems are parallel to (011) and molecules are connected *via* inter­molecular N—H⋯N hydrogen bonds, forming chains along [100]. The crystal studied was an inversion twin with a 0.86 (5):0.14 (5) domain ratio.

## Related literature

For background to quinoline compounds, see: Elderfield (1960 ▶); Wright *et al.* (2001 ▶); Sahu *et al.* (2002 ▶); Bringmann *et al.* (2004 ▶); Kournetsov *et al.* (2005 ▶). For the biological and pharmaceutical applications of quinolines, see: Albert & Ritchie (1955 ▶); Mouzine *et al.* (1980 ▶); Lyle & Keefer (1967 ▶). For the general synthesis of quinolines, see: Cope & Ciganek (1963 ▶); Ohta *et al.* (1989 ▶); Hatanaka & Ojima (1981 ▶); Smith (1994 ▶); Borch *et al.* (1971 ▶). For related structures, see: Boulcina *et al.* (2007 ▶, 2008 ▶).
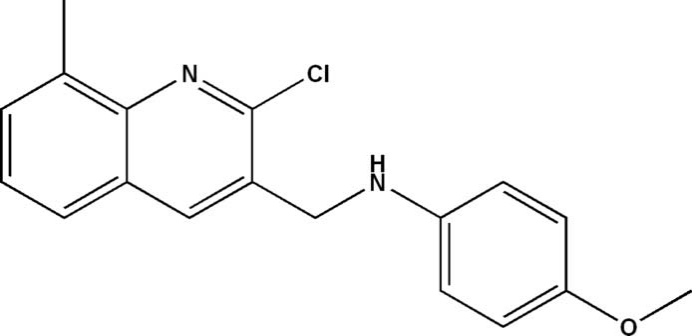


## Experimental

### 

#### Crystal data

C_18_H_17_ClN_2_O
*M* *_r_* = 312.79
Orthorhombic, 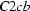

*a* = 7.3067 (1) Å
*b* = 17.7803 (4) Å
*c* = 22.8221 (5) Å
*V* = 2964.94 (10) Å^3^
*Z* = 8
Mo *K*α radiation
μ = 0.26 mm^−1^
*T* = 100 K
0.41 × 0.29 × 0.17 mm

#### Data collection

Bruker APEXII diffractometer
Absorption correction: multi-scan (*SADABS*; Sheldrick, 2002 ▶) *T*_min_ = 0.857, *T*_max_ = 0.957
15861 measured reflections
3383 independent reflections
3303 reflections with *I* > 2σ(*I*)
*R*_int_ = 0.030

#### Refinement

*R*[*F* ^2^ > 2σ(*F* ^2^)] = 0.027
*wR*(*F* ^2^) = 0.070
*S* = 1.05
3383 reflections
205 parameters
1 restraint
H atoms treated by a mixture of independent and constrained refinement
Δρ_max_ = 0.30 e Å^−3^
Δρ_min_ = −0.17 e Å^−3^
Absolute structure: Flack (1983 ▶), 1557 Friedel pairs
Flack parameter: 0.14 (5)

### 

Data collection: *APEX2* (Bruker,2001 ▶); cell refinement: *SAINT* (Bruker, 2001 ▶); data reduction: *SAINT*; program(s) used to solve structure: *SIR2002* (Burla *et al.*, 2003 ▶); program(s) used to refine structure: *SHELXL97* (Sheldrick, 2008 ▶); molecular graphics: *ORTEP-3 for Windows* (Farrugia, 1997 ▶) and *DIAMOND* (Brandenburg & Berndt, 2001 ▶); software used to prepare material for publication: *WinGX* publication routines (Farrugia, 1999 ▶).

## Supplementary Material

Crystal structure: contains datablocks global, I. DOI: 10.1107/S1600536810041061/lh5139sup1.cif

Structure factors: contains datablocks I. DOI: 10.1107/S1600536810041061/lh5139Isup2.hkl

Additional supplementary materials:  crystallographic information; 3D view; checkCIF report

## Figures and Tables

**Table 1:** Hydrogen-bond geometry (Å, °)

*D*—H⋯*A*	*D*—H	H⋯*A*	*D*⋯*A*	*D*—H⋯*A*
N13—H13*N*⋯N2^i^	0.82 (3)	2.56 (3)	3.3471 (16)	163 (2)

Symmetry code: (i) 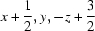.

**Fig. 1.**
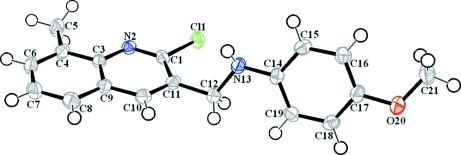
The molecular structure of the title compound with the atomic labelling scheme. Displacement are drawn at the 50% probability level.

**Fig. 2.**
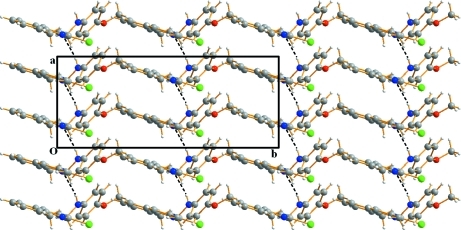
Part of the of the layered crystal packing of (I) viewed along the c axis (hydrogen bonds [N—H···N] are shown as dashed line between layers).

## supplementary crystallographic information

### Comment

The importance of quinoline and its derivatives is well recognized by synthetic and biological chemists (Elderfield *et al.*, 1960; Wright *et al.*, 2001; Sahu *et al.*, 2002; Bringmann *et al.*, 2004; Kournetsov *et al.*, 2005). Compounds possessing this ring system such as aminoquinolines, have wide applications as drugs and pharmaceuticals. Many derivatives of aminoquinolines have been reported as plant resistance factors as topical antiseptic (Albert *et al.* 1955), analgesic (Mouzine *et al.*, 1980) and antimalarials (Lyle *et al.*, 1967). Therefore, considerable efforts have been directed towards the preparation and synthetic manipulation of these molecules. Of the many methods available for the synthesis of amines, the most widely used is the reduction of amides (Cope *et al.*, 1963) and nitro compounds (Ohta *et al.*, 1989) with LiAlH_4_. Reduction of azides (Hatanaka *et al.*, 1981) and nitro compounds (Smith, 1994) with H_2_/Pd have been also reported. Another important and simple method has been disclosed by Borch *et al.* (1971) which employs NaBH_3_CN at pH≈6 to reduce imines to the corresponding amines. In an ongoing project in our laboratory based on the synthesis of functionalized quinolines (Boulcina *et al.*, 2007; 2008), we require an efficient route for the synthesis and transformations of these heterocycles. Herein, we report an efficient and general procedure for the synthesis of a new aminoquinoline derivative derived from 2-chloro-8-methyl-3-formylquinolines. The use of NaBH_3_CN as a reducing agent of the corresponding imines was the methodology of choice to accomplish this task. The crystal structure of the title compound (I) is determined herein.

The molecular structure and the atom-numbering scheme of (I) are shown in Fig. 1. The quinoline ring system is essentially planar; the rms deviation for the non-H atoms is 0.04 Å with a maximum deviation from the mean plane of -0.026 (4)Å for the C atom bonded to the -CH_2_- group. The methoxy substituted benzene ring forms a dihedral of 70.22 (4)° with this ring system. The crystal structure can be described as zig-zag layers in which the quinoline ring systems are parallel to the (011) plane and moleclues are connected via intermolecular N—H···N hydrogen bond forming chains along [100]. The crystal is an inversion twin with a 0.86 (5):0.14 (5) ratio of domains.

### Experimental

A mixture of 2-chloro-8-methyl-3-formylquinoline (5 mmol) and 4-methoxyaniline (5 mmol) in methanol (10 ml) was stirred at ambient temperature. On completion of the reaction, as indicated by TLC, the mixture was then filtered and the resulting product was washed with cold methanol. The product thus obtained as slightly yellow powder, could be used in the next step without purification. Further recrystallization from methanol yields pure imine. The appropriate imine (1 mmol) and NaBH_3_CN (3 mmol) in methanol (10 ml) were stirred for 24 h, diluted with cold water (20 ml), and left for several hours. The resulting solid was filtered off, washed with water, then with ethanol and with hexane. *N*-((2-chloro-8-methylquinolin-3-yl)methyl)-4-methoxybenzenamine was recrystallized from ethanol and identified by IR, ^1^H and ^13^C NMR spectroscopies. Crystals of the title compound were obtained by slow crystallization from a methanol solution.

### Refinement

All H atoms were vizible in difference Fourier maps but were introduced in calculated positions and treated as riding on their parent C atom (with C—H = 0.93–0.97Å and *U*_iso_(H) =1.2 or 1.5(U_eq_carrier atom)), except for H13N which was located in a difference Fourier map and refined isotropically.

### Crystal data

**Table:**

C_18_H_17_ClN_2_O	*F*(000) = 1312
*M**_r_* = 312.79	*D*_x_ = 1.401 Mg m^−^^3^
Orthorhombic, *C*2*c**b*	Mo *K*α radiation, λ = 0.71073 Å
Hall symbol: C -2bc 2	Cell parameters from 9705 reflections
*a* = 7.3067 (1) Å	θ = 2.3–27.4°
*b* = 17.7803 (4) Å	µ = 0.26 mm^−^^1^
*c* = 22.8221 (5) Å	*T* = 100 K
*V* = 2964.94 (10) Å^3^	Prism, colourless
*Z* = 8	0.41 × 0.29 × 0.17 mm

### Data collection

**Table:**

Bruker APEXII diffractometer	3303 reflections with *I* > 2σ(*I*)
graphite	*R*_int_ = 0.030
CCD rotation images, thin slices scans	θ_max_ = 27.4°, θ_min_ = 3.1°
Absorption correction: multi-scan (*SADABS*; Sheldrick, 2002)	*h* = −9→9
*T*_min_ = 0.857, *T*_max_ = 0.957	*k* = −22→22
15861 measured reflections	*l* = −29→29
3383 independent reflections	

### Refinement

**Table:**

Refinement on *F*^2^	Secondary atom site location: difference Fourier map
Least-squares matrix: full	Hydrogen site location: inferred from neighbouring sites
*R*[*F*^2^ > 2σ(*F*^2^)] = 0.027	H atoms treated by a mixture of independent and constrained refinement
*wR*(*F*^2^) = 0.070	*w* = 1/[σ^2^(*F*_o_^2^) + (0.0402*P*)^2^ + 1.310*P*] where *P* = (*F*_o_^2^ + 2*F*_c_^2^)/3
*S* = 1.05	(Δ/σ)_max_ = 0.001
3383 reflections	Δρ_max_ = 0.30 e Å^−^^3^
205 parameters	Δρ_min_ = −0.17 e Å^−^^3^
1 restraint	Absolute structure: Flack (1983), 1557 Friedel pairs
Primary atom site location: structure-invariant direct methods	Flack parameter: 0.14 (5)

### Special details

**Table:**

Geometry. All e.s.d.'s (except the e.s.d. in the dihedral angle between two l.s. planes) are estimated using the full covariance matrix. The cell e.s.d.'s are taken into account individually in the estimation of e.s.d.'s in distances, angles and torsion angles; correlations between e.s.d.'s in cell parameters are only used when they are defined by crystal symmetry. An approximate (isotropic) treatment of cell e.s.d.'s is used for estimating e.s.d.'s involving l.s. planes.
Refinement. Refinement of *F*^2^ against ALL reflections. The weighted *R*-factor *wR* and goodness of fit *S* are based on *F*^2^, conventional *R*-factors *R* are based on *F*, with *F* set to zero for negative *F*^2^. The threshold expression of *F*^2^ > σ(*F*^2^) is used only for calculating *R*-factors(gt) *etc*. and is not relevant to the choice of reflections for refinement. *R*-factors based on *F*^2^ are statistically about twice as large as those based on *F*, and *R*- factors based on ALL data will be even larger.

### Fractional atomic coordinates and isotropic or equivalent isotropic displacement parameters (Å^2^)

**Table:**

	*x*	*y*	*z*	*U*_iso_*/*U*_eq_	
Cl1	0.66000 (6)	0.143865 (16)	0.747584 (13)	0.01770 (8)	
C1	0.72948 (19)	0.05005 (8)	0.75340 (5)	0.0142 (3)	
N2	0.74170 (16)	0.02403 (6)	0.80680 (5)	0.0146 (2)	
C3	0.79448 (18)	−0.04968 (7)	0.81373 (6)	0.0140 (2)	
C4	0.80333 (18)	−0.08019 (8)	0.87155 (6)	0.0161 (3)	
C5	0.7535 (2)	−0.03278 (8)	0.92397 (6)	0.0218 (3)	
H5A	0.6239	−0.0240	0.9242	0.033*	
H5B	0.8168	0.0145	0.9219	0.033*	
H5C	0.7881	−0.0586	0.9592	0.033*	
C6	0.85641 (19)	−0.15425 (8)	0.87761 (6)	0.0187 (3)	
H6	0.8633	−0.1746	0.9151	0.022*	
C7	0.9006 (2)	−0.20031 (8)	0.82926 (6)	0.0206 (3)	
H7	0.9356	−0.2500	0.8352	0.025*	
C8	0.8918 (2)	−0.17158 (8)	0.77354 (6)	0.0187 (3)	
H8	0.9215	−0.2017	0.7416	0.022*	
C9	0.83755 (19)	−0.09606 (8)	0.76480 (6)	0.0150 (3)	
C10	0.81977 (18)	−0.06374 (8)	0.70812 (6)	0.0161 (3)	
H10	0.8462	−0.0928	0.6753	0.019*	
C11	0.76461 (18)	0.00913 (8)	0.70091 (5)	0.0152 (3)	
C12	0.7358 (2)	0.04066 (8)	0.64019 (5)	0.0171 (3)	
H12A	0.7357	−0.0003	0.6121	0.021*	
H12B	0.6170	0.0649	0.6384	0.021*	
N13	0.87651 (16)	0.09478 (6)	0.62391 (5)	0.0162 (2)	
C14	0.87924 (19)	0.12075 (7)	0.56569 (5)	0.0143 (3)	
C15	0.74893 (19)	0.09871 (7)	0.52389 (6)	0.0161 (3)	
H15	0.6604	0.0634	0.5338	0.019*	
C16	0.75084 (19)	0.12924 (8)	0.46776 (6)	0.0164 (3)	
H16	0.6633	0.1141	0.4406	0.020*	
C17	0.88177 (19)	0.18205 (7)	0.45168 (5)	0.0159 (3)	
C18	1.01471 (19)	0.20355 (8)	0.49223 (6)	0.0171 (3)	
H18	1.1045	0.2381	0.4818	0.021*	
C19	1.01231 (18)	0.17304 (8)	0.54846 (6)	0.0164 (3)	
H19	1.1013	0.1878	0.5753	0.020*	
O20	0.86737 (15)	0.21049 (6)	0.39555 (4)	0.0202 (2)	
C21	0.9605 (2)	0.27960 (9)	0.38392 (6)	0.0220 (3)	
H21A	0.9370	0.3145	0.4151	0.033*	
H21B	0.9173	0.3003	0.3476	0.033*	
H21C	1.0897	0.2704	0.3812	0.033*	
H13N	0.978 (4)	0.0849 (14)	0.6370 (11)	0.050*	

### Atomic displacement parameters (Å^2^)

**Table:**

	*U*^11^	*U*^22^	*U*^33^	*U*^12^	*U*^13^	*U*^23^
Cl1	0.02077 (15)	0.01491 (14)	0.01743 (14)	0.00316 (14)	0.00039 (13)	0.00172 (12)
C1	0.0131 (6)	0.0119 (6)	0.0177 (6)	−0.0001 (5)	0.0003 (5)	0.0017 (5)
N2	0.0130 (5)	0.0160 (5)	0.0146 (5)	−0.0004 (4)	−0.0006 (4)	−0.0001 (4)
C3	0.0122 (6)	0.0152 (6)	0.0147 (5)	−0.0019 (5)	−0.0010 (5)	0.0015 (5)
C4	0.0153 (6)	0.0188 (6)	0.0143 (6)	−0.0008 (5)	−0.0016 (5)	0.0011 (5)
C5	0.0307 (8)	0.0225 (7)	0.0121 (6)	0.0003 (6)	−0.0002 (6)	0.0014 (5)
C6	0.0178 (6)	0.0210 (7)	0.0172 (6)	−0.0019 (5)	−0.0030 (5)	0.0062 (5)
C7	0.0208 (6)	0.0161 (6)	0.0249 (7)	0.0008 (6)	−0.0036 (6)	0.0033 (5)
C8	0.0194 (7)	0.0165 (6)	0.0202 (6)	0.0002 (6)	0.0006 (6)	−0.0018 (5)
C9	0.0134 (6)	0.0156 (6)	0.0159 (6)	−0.0018 (5)	−0.0004 (5)	0.0011 (5)
C10	0.0156 (6)	0.0183 (6)	0.0143 (6)	−0.0024 (5)	0.0015 (5)	−0.0013 (5)
C11	0.0138 (6)	0.0185 (7)	0.0133 (6)	−0.0027 (5)	−0.0002 (5)	0.0004 (5)
C12	0.0204 (6)	0.0178 (6)	0.0131 (6)	−0.0035 (5)	−0.0009 (5)	0.0020 (5)
N13	0.0162 (6)	0.0189 (6)	0.0134 (5)	−0.0011 (5)	−0.0011 (4)	0.0022 (4)
C14	0.0175 (7)	0.0135 (5)	0.0120 (6)	0.0032 (5)	0.0013 (5)	−0.0005 (5)
C15	0.0173 (6)	0.0140 (6)	0.0169 (6)	−0.0018 (5)	0.0012 (5)	−0.0004 (5)
C16	0.0175 (7)	0.0164 (6)	0.0153 (6)	0.0006 (5)	−0.0021 (5)	−0.0032 (5)
C17	0.0192 (6)	0.0164 (6)	0.0120 (6)	0.0026 (6)	0.0013 (5)	0.0006 (5)
C18	0.0174 (6)	0.0188 (7)	0.0152 (6)	−0.0016 (5)	0.0022 (5)	0.0003 (5)
C19	0.0158 (6)	0.0195 (7)	0.0140 (6)	−0.0005 (5)	−0.0009 (5)	−0.0021 (5)
O20	0.0276 (6)	0.0208 (5)	0.0122 (4)	−0.0029 (4)	−0.0006 (4)	0.0022 (4)
C21	0.0216 (7)	0.0249 (7)	0.0194 (7)	−0.0013 (6)	0.0026 (5)	0.0070 (6)

### Geometric parameters (Å, °)

**Table:**

Cl1—C1	1.7486 (14)	C12—N13	1.4566 (18)
C1—N2	1.3067 (16)	C12—H12A	0.9700
C1—C11	1.4249 (18)	C12—H12B	0.9700
N2—C3	1.3752 (18)	N13—C14	1.4068 (16)
C3—C9	1.4236 (19)	N13—H13N	0.82 (3)
C3—C4	1.4281 (18)	C14—C19	1.4016 (19)
C4—C6	1.3795 (19)	C14—C15	1.4036 (19)
C4—C5	1.5082 (19)	C15—C16	1.3913 (18)
C5—H5A	0.9600	C15—H15	0.9300
C5—H5B	0.9600	C16—C17	1.390 (2)
C5—H5C	0.9600	C16—H16	0.9300
C6—C7	1.411 (2)	C17—O20	1.3812 (15)
C6—H6	0.9300	C17—C18	1.395 (2)
C7—C8	1.3718 (19)	C18—C19	1.3935 (19)
C7—H7	0.9300	C18—H18	0.9300
C8—C9	1.4143 (19)	C19—H19	0.9300
C8—H8	0.9300	O20—C21	1.4294 (18)
C9—C10	1.4213 (19)	C21—H21A	0.9600
C10—C11	1.3669 (19)	C21—H21B	0.9600
C10—H10	0.9300	C21—H21C	0.9600
C11—C12	1.5097 (17)		
			
N2—C1—C11	126.23 (13)	N13—C12—C11	112.39 (11)
N2—C1—Cl1	115.37 (10)	N13—C12—H12A	109.1
C11—C1—Cl1	118.40 (10)	C11—C12—H12A	109.1
C1—N2—C3	117.64 (11)	N13—C12—H12B	109.1
N2—C3—C9	121.59 (12)	C11—C12—H12B	109.1
N2—C3—C4	118.75 (12)	H12A—C12—H12B	107.9
C9—C3—C4	119.65 (12)	C14—N13—C12	117.89 (11)
C6—C4—C3	117.90 (12)	C14—N13—H13N	113.6 (18)
C6—C4—C5	121.45 (12)	C12—N13—H13N	114.0 (18)
C3—C4—C5	120.64 (12)	C19—C14—C15	117.72 (12)
C4—C5—H5A	109.5	C19—C14—N13	119.50 (12)
C4—C5—H5B	109.5	C15—C14—N13	122.73 (12)
H5A—C5—H5B	109.5	C16—C15—C14	120.67 (12)
C4—C5—H5C	109.5	C16—C15—H15	119.7
H5A—C5—H5C	109.5	C14—C15—H15	119.7
H5B—C5—H5C	109.5	C17—C16—C15	120.90 (12)
C4—C6—C7	122.67 (13)	C17—C16—H16	119.5
C4—C6—H6	118.7	C15—C16—H16	119.5
C7—C6—H6	118.7	O20—C17—C16	116.09 (12)
C8—C7—C6	119.86 (13)	O20—C17—C18	124.60 (12)
C8—C7—H7	120.1	C16—C17—C18	119.30 (12)
C6—C7—H7	120.1	C19—C18—C17	119.70 (13)
C7—C8—C9	119.83 (13)	C19—C18—H18	120.1
C7—C8—H8	120.1	C17—C18—H18	120.1
C9—C8—H8	120.1	C18—C19—C14	121.69 (12)
C8—C9—C10	122.55 (13)	C18—C19—H19	119.2
C8—C9—C3	120.08 (12)	C14—C19—H19	119.2
C10—C9—C3	117.36 (12)	C17—O20—C21	116.79 (11)
C11—C10—C9	121.32 (13)	O20—C21—H21A	109.5
C11—C10—H10	119.3	O20—C21—H21B	109.5
C9—C10—H10	119.3	H21A—C21—H21B	109.5
C10—C11—C1	115.84 (12)	O20—C21—H21C	109.5
C10—C11—C12	120.24 (11)	H21A—C21—H21C	109.5
C1—C11—C12	123.85 (12)	H21B—C21—H21C	109.5
			
C11—C1—N2—C3	0.4 (2)	N2—C1—C11—C10	−1.4 (2)
Cl1—C1—N2—C3	179.51 (10)	Cl1—C1—C11—C10	179.53 (10)
C1—N2—C3—C9	1.04 (19)	N2—C1—C11—C12	175.65 (14)
C1—N2—C3—C4	−178.05 (13)	Cl1—C1—C11—C12	−3.40 (19)
N2—C3—C4—C6	179.95 (12)	C10—C11—C12—N13	−108.76 (14)
C9—C3—C4—C6	0.83 (19)	C1—C11—C12—N13	74.30 (17)
N2—C3—C4—C5	0.7 (2)	C11—C12—N13—C14	171.73 (11)
C9—C3—C4—C5	−178.37 (13)	C12—N13—C14—C19	179.78 (13)
C3—C4—C6—C7	−0.4 (2)	C12—N13—C14—C15	2.33 (19)
C5—C4—C6—C7	178.83 (14)	C19—C14—C15—C16	−1.26 (19)
C4—C6—C7—C8	0.1 (2)	N13—C14—C15—C16	176.24 (12)
C6—C7—C8—C9	−0.3 (2)	C14—C15—C16—C17	0.2 (2)
C7—C8—C9—C10	−177.82 (13)	C15—C16—C17—O20	−177.93 (12)
C7—C8—C9—C3	0.8 (2)	C15—C16—C17—C18	1.1 (2)
N2—C3—C9—C8	179.84 (13)	O20—C17—C18—C19	177.70 (12)
C4—C3—C9—C8	−1.1 (2)	C16—C17—C18—C19	−1.3 (2)
N2—C3—C9—C10	−1.44 (19)	C17—C18—C19—C14	0.1 (2)
C4—C3—C9—C10	177.64 (12)	C15—C14—C19—C18	1.1 (2)
C8—C9—C10—C11	179.08 (14)	N13—C14—C19—C18	−176.46 (12)
C3—C9—C10—C11	0.40 (19)	C16—C17—O20—C21	161.27 (12)
C9—C10—C11—C1	0.91 (19)	C18—C17—O20—C21	−17.73 (19)
C9—C10—C11—C12	−176.27 (13)		

### Hydrogen-bond geometry (Å, °)

**Table:**

*D*—H···*A*	*D*—H	H···*A*	*D*···*A*	*D*—H···*A*
N13—H13N···N2^i^	0.82 (3)	2.56 (3)	3.3471 (16)	163 (2)

Symmetry codes: (i) *x*+1/2, *y*, −*z*+3/2.
